# Impact of the COVID-19 Pandemic on Functionality and Fall Risk in Institutionalized Geriatric Patients: A Longitudinal Observational Study

**DOI:** 10.3390/life15071130

**Published:** 2025-07-18

**Authors:** Javier Torralba Estelles, Jorge Velert Belenguer, Elena Martinez Mendoza, Javier Ferrer Torregrosa

**Affiliations:** 1 Department of Podiatry, School of Medicine and Health Science, Catholic University of Valencia, 46900 Torrent, PC, Spain; 2 Private Practice at dePie Clínicas Podológicas, 46021 Valencia, PC, Spain

**Keywords:** elderly, COVID-19, functional independence, balance, gait, Tinetti test, Barthel test

## Abstract

Background: The global impact of the COVID-19 pandemic has significantly influenced elderly functionality, particularly in terms of balance, gait, and independence in daily activities. This study sought to evaluate how these aspects have changed over the course of the health crisis. Methods: We employed the Tinetti scale for assessing balance and gait, and the Barthel Index for measuring functional independence, conducting a comparative analysis of scores before and after the onset of the pandemic in a sample of elderly individuals. Results: Our findings indicated an increase in Tinetti scores, suggesting some improvement in balance and mobility, albeit with marked variability across participants. On the other hand, Barthel scores showed a significant decline, pointing to a reduction in functional independence. Conclusions: These results suggest that the impact of COVID-19 on elderly functionality is not uniform, highlighting the need for personalized rehabilitation strategies. Such strategies should not only focus on physical recovery but also consider the psychological and social repercussions of the pandemic to fully address the diverse needs of this vulnerable population.

## 1. Introduction

Falls among the geriatric population represent a major public health concern, as they are the leading cause of injury-related morbidity [1] and rank seventh among causes of mortality [2] in individuals over the age of 65. It is estimated that approximately one-third of older adults experience at least one fall per year [1], leading to a variety of physical, psychological, and social consequences. Among the most common adverse outcomes are bone fractures [3,4], loss of autonomy, and the development of fear of falling again, which may result in a significant reduction in mobility and increased sedentary behavior. This cycle of decreased physical activity and functional decline not only deteriorates the quality of life in older adults but also increases the burden on healthcare systems and long-term care services.

Multiple factors contribute to fall risk in older adults, including sarcopenia, reduced muscular strength, gait and balance disturbances, cognitive impairment, polypharmacy, and the presence of chronic conditions such as hypertension, diabetes, and osteoporosis [5]. Furthermore, frailty—a condition characterized by decreased physiological reserve and increased vulnerability to adverse events—is strongly associated with fall risk and a diminished capacity for recovery following injury [6]. Validated tools used to assess functional independence and predict fall risk in older adults include the Tinetti Performance-Oriented Mobility Assessment, which evaluates balance and gait [5], and the Barthel Index, which measures functional ability in activities of daily living [7].

The COVID-19 pandemic has posed an unprecedented challenge to the health and well-being of the elderly. Beyond the high risk of complications and mortality associated with SARS-CoV-2 infection in this population, the implementation of lockdown and social distancing measures has had a detrimental impact on functionality and mobility [8]. Several studies have reported increased sedentary behavior, decreased physical activity, and muscle strength deterioration among older adults during the pandemic, resulting in higher prevalence of frailty and increased fall risk [9,10]. In Finland, for instance, a decrease in community mobility and increased social isolation in older adults were observed after the onset of the pandemic [11].

Prolonged social isolation has further contributed to reduced physical activity, accelerating muscle mass loss and motor dysfunction in older adults [12]. Exercise programs involving strength, balance, and flexibility training have been shown to be effective in preventing frailty and reducing fall risk in this population [13]. However, the interruption of such programs due to public health restrictions has led to significant functional decline in many older individuals [8].

In addition to physical effects, the pandemic has had substantial psycho-affective impacts on the elderly, including increased anxiety, depression, and feelings of loneliness [8]. These factors, combined with decreased mobility, may have further heightened fall risk by impairing motor coordination, decision-making capacity, and self-confidence in performing daily activities [9].

Although previous studies have examined falls in geriatric populations, there remains limited evidence on the specific impact of the pandemic on functionality and fall risk [10]. Understanding how the public health crisis has affected these aspects is critical to developing preventive and rehabilitative strategies aimed at mitigating its long-term effects and informing protocols for future similar scenarios.

The primary objective of this study is to assess the impact of the COVID-19 pandemic on functionality and fall risk in institutionalized geriatric patients through comparative analysis of Tinetti and Barthel test scores before and after the pandemic.

Our hypothesis is that the COVID-19 pandemic negatively impacted the overall functionality of institutionalized geriatric patients, particularly affecting independence in activities of daily living due to prolonged isolation and restrictions.

## 2. Materials and Methods

### 2.1. Study Desing

A longitudinal observational study was conducted to analyze changes in functionality and fall risk among institutionalized geriatric patients before and after the COVID-19 pandemic. A comparative analysis was performed on the scores obtained from the Tinetti Performance-Oriented Mobility Assessment and the Barthel Index during both time periods, in order to evaluate the impact of the pandemic on participants’ mobility and functional autonomy. The pre-COVID-19 assessment was conducted between October and December 2019, being the last functional evaluation performed immediately before lockdown measures, and the post-COVID-19 assessment was conducted between March and May 2023, immediately after restrictions were lifted. The relatively long interval of approximately 3.5 years between assessments could independently contribute to functional and mobility changes, thus limiting our ability to attribute observed outcomes exclusively to the pandemic.

### 2.2. Participants

The study included older adults of both sexes institutionalized in geriatric care facilities. Participants were selected based on having provided signed informed consent and residing in facilities operated by the Generalitat Valenciana (see Table 1). Residents had access to structured physical activity programs, although participation varied depending on individual health status and personal willingness. All residents received routine support from caregiving staff for basic daily living activities, although the level of assistance provided was adjusted according to individual needs and functional independence.

### 2.3. Inclusion and Exclusion Criteria

Inclusion Criteria

Institutionalized geriatric patients (≥65 years) residing in long-term care facilities.

Informed consent provided by the patient or their legal representative.

Exclusion Criteria

Patients with advanced neurodegenerative diseases (e.g., severe-stage Alzheimer’s disease, advanced Parkinson’s disease).

Subjects with severe immobility.

### 2.4. Variables

The variables evaluated included sex, age, height, and weight. The Body Mass Index (BMI) was calculated and categorized according to the World Health Organization classification.

The primary assessments used were the Tinetti Performance-Oriented Mobility Assessment and the Barthel Index.

The Tinetti Test is an evaluation tool designed to assess fall risk in older adults, focusing on two fundamental components: balance and gait. To evaluate static balance, the test observes abilities such as rising from a chair without assistance, maintaining a stable standing position, closing the eyes while remaining balanced, turning the head, and completing a full 360-degree turn. Gait analysis includes initiation of walking, step length and height, symmetry, path deviation, walking speed, and the ability to stop safely. The maximum total score is 28 points, with a score below 19 indicating a high risk of falls. This test not only identifies general fall risk but also highlights specific areas requiring intervention to improve stability and mobility [14,15].

The Barthel Index [16], also used as a primary outcome measure in this study, is a scale designed to assess an individual’s ability to perform basic activities of daily living (ADLs), thereby providing an evaluation of functional independence. It includes several activities such as feeding, bathing, grooming, dressing, bowel and bladder control, transferring from bed to chair, bed mobility, wheelchair management if applicable, and stair climbing. Each item is assigned a specific score, contributing to a maximum total of 100 points. Higher scores indicate greater independence, whereas lower scores reflect increased dependency in daily life. The Barthel Index is useful not only for evaluating the current functional status of a patient but also for monitoring changes over time, thus supporting care planning and rehabilitation strategies.

### 2.5. Statistical Analisys

A descriptive analysis was conducted, calculating means and standard deviations, as well as frequencies for the variables of interest. Normality of distribution for pre- and post-COVID-19 scores on the Tinetti and Barthel tests was assessed using the Shapiro–Wilk test. Based on the results, comparisons between pre- and post-COVID-19 periods were made using the paired Student’s *t*-test for normally distributed data (*p* > 0.05), or the Wilcoxon signed-rank test for non-normally distributed data (*p* < 0.05).

Effect size was interpreted using the following thresholds: trivial (<0.20), small (0.20–0.59), moderate (0.60–1.19), large (1.20–1.99), and very large (>2.00) [17].

Sex-based differences in score evolution were analyzed using the Mann–Whitney U test or Student’s *t*-test, depending on data normality.

A significance level of *p* > 0.05 was established. Statistical analyses were performed using SPSS software (version 24; SPSS Inc., Chicago, IL, USA), and graphical representations were generated using JASP software (version 0.16.4; Amsterdam, The Netherlands).

## 3. Results

The assessment of Tinetti and Barthel scores before and after the COVID-19 pandemic revealed significant differences in participants’ balance, gait, and functional independence. The pre-COVID-19 Tinetti score had a mean of 20.95 with a standard deviation of 19.75. Post-COVID-19, the mean increased to 24.51, accompanied by greater data dispersion, as evidenced by a standard deviation of 27.27 and a coefficient of variation of 1.11. This increase suggests greater variability in post-pandemic responses, potentially associated with individual factors such as rehabilitation access, prior health status, and the impact of lockdown measures on mobility.

In contrast, the Barthel Index, used to evaluate functional independence, showed an opposite trend. Prior to the pandemic, the mean score was 53.16 with a standard deviation of 33.53. After the pandemic, the mean decreased to 44.89, with a standard deviation of 33.50. Although data dispersion remained stable, the coefficient of the variation increased from 0.63 to 0.75, indicating greater heterogeneity in post-COVID-19 outcomes. This decline suggests that participants’ functional capacity was negatively affected, likely due to reduced physical activity, the impact of viral infection on motor function, and possibly limited access to rehabilitation services during the pandemic.

Table 2 presents the results of the paired comparison using the Wilcoxon signed-rank test, which was applied to compare pre- and post-COVID-19 scores on the Tinetti and Barthel scales. This analysis evaluates whether significant differences exist between both time points and the magnitude of those changes.

For the Tinetti score, the test statistic WWW represents the sum of ranks, with a z-value of 2.79, indicating a statistically significant difference between pre- and post-COVID-19 measurements. The *p*-value of 0.00511 confirms that this difference is unlikely due to chance, with a significance level below 0.01. However, the rank–biserial correlation was 0.21, suggesting a small-to-moderate effect size. The standard error of the correlation was 0.07, indicating an acceptable margin of error in estimating the relationship between measurements. These findings suggest that although there was a significant change in Tinetti scores post-COVID-19, the magnitude of the difference was not particularly large and may vary substantially among individuals.

In contrast, the Barthel score exhibited a larger WWW value compared to Tinetti, indicating a more pronounced difference between pre- and post-COVID-19 scores. The z-value was 10.34, substantially higher than that for Tinetti, reflecting a stronger impact on participants’ functionality. The *p*-value was <0.001, denoting a highly significant difference. Additionally, the rank–biserial correlation was 0.69, indicating a large effect size and thus a more marked impairment in functional independence. The standard error of the correlation was 0.07, reflecting a precise estimation.

**Figure 1:**
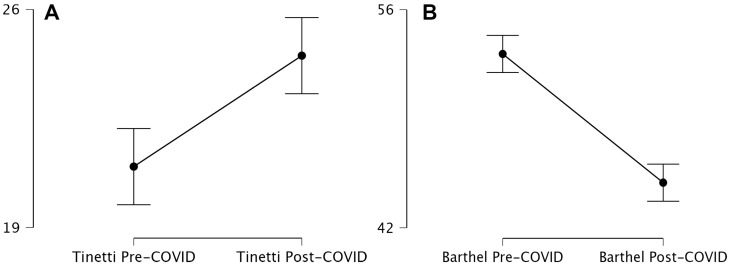
Tinetti and Barthel scores in geriatric patients (2019–2023). Descriptives plot. Tinetti (**A**) and Barthel (**B**) scores in geriatric patients (2019–2023).

Overall, these findings indicate that COVID-19 had a significant impact on the functional status of the participants. While Tinetti scores demonstrated a statistically significant change with a relatively small effect size—pointing to greater individual variability—the Barthel Index showed a considerable decline with a large effect size, indicating a more homogeneous and severe deterioration in functional independence. These results highlight the urgent need to implement individualized rehabilitation strategies to mitigate post-COVID-19 sequelae and improve the quality of life in affected elderly populations (Figure 1).

In the case of the Tinetti score, the obtained WWW statistic represents the sum of ranks in the test, and the z-value was 2.79, suggesting a statistically significant difference between pre- and post-COVID-19 measurements. The corresponding *p*-value of 0.00511 confirms that this difference is unlikely to be due to chance, with a significance level below 0.01. However, the rank–biserial correlation was 0.21, indicating a small to moderate effect size. The standard error of the correlation was 0.07, suggesting an acceptable margin of error in estimating the relationship between the two measurements. These results indicate that although there was a significant change in the Tinetti score following COVID-19, the magnitude of this difference was not particularly large and may vary considerably among individuals.

In contrast, the Barthel score presented a higher WWW value compared to Tinetti, indicating a more pronounced difference between pre- and post-COVID-19 measurements. The z-value was 10.34, substantially greater than that observed for Tinetti, reflecting a stronger impact on participants’ functional status. The *p*-value was <0.001, demonstrating a highly significant difference. Moreover, the rank–biserial correlation was 0.69, indicating a large effect size, and thus a more evident decline in functional independence. The standard error of the correlation was 0.07, confirming a precise estimation.

These findings clearly show that COVID-19 had a significant impact on participants’ functionality. While the Tinetti score exhibited a statistically significant change with a smaller effect size—suggesting greater individual variability—the Barthel score showed a substantial decline with a large effect size, reflecting a more homogeneous and severe deterioration in functional independence. These outcomes emphasize the critical need to implement personalized rehabilitation strategies to mitigate post-COVID-19 sequelae and enhance the quality of life in affected elderly individuals.

### 3.1. Influence of Gender on Functional Recovery

The analysis of the delta variable—defined as the difference between pre- and post-COVID-19 scores—for the Tinetti test by gender revealed a Wilcoxon test statistic W = 21,341.00 W = 21,341.00 W = 21,341.00 with a *p*-value of 0.60, indicating no statistically significant difference in score variation between males and females. The rank–biserial correlation was −0.03, suggesting a null effect, which implies that gender did not influence the improvement or deterioration in balance and gait as measured by the Tinetti test following COVID-19.

Similarly, the comparison of changes in the Barthel Index scores yielded a W = 22,078.00 W = 22,078.00 W = 22,078.00 with a *p*-value of 0.89, confirming the absence of significant differences in the recovery of functional independence between genders. The rank–biserial correlation was 0.06, reflecting a negligible effect.

These results suggest that other factors—such as prior health status, disease severity, or access to rehabilitation services—may be more decisive in determining functional recovery than biological sex.

### 3.2. Influence of Body Mass Index on Improvement in Functional Test Performance

An analysis of variance (ANOVA) was performed to assess the relationship between Body Mass Index (BMI) classification and improvement in the Tinetti and Barthel functional tests. The analysis revealed no statistically significant differences among BMI groups.

For improvement in the Tinetti test, the ANOVA yielded an F-value of 1.85 with a *p*-value of 0.14, indicating that BMI classification did not have a significant effect on balance and gait recovery. Descriptive statistics showed that the underweight group had the highest mean improvement (7.79), followed by the obese (5.53), normal weight (2.97), and overweight (1.24) groups. However, post hoc comparisons did not detect statistically significant differences between groups, suggesting that BMI is not a determining factor in mobility recovery as assessed by the Tinetti scale.

Regarding the Barthel Index, the ANOVA produced an F-value of 0.21 with a *p*-value of 0.89, again indicating that BMI classification did not influence the recovery of functional independence. Descriptive values showed similar mean changes across all BMI groups, with negative values indicating a decline in functionality, but without significant differences between BMI categories.

The effect size of BMI on improvement in functional test scores was very small. For the Tinetti test, η^2^ = 0.01, meaning only 1% of the variability in improvement could be attributed to BMI. For the Barthel Index, η^2^ = 1.43 × 10^−3^, indicating a negligible influence.

## 4. Discussion

The results suggest that while mobility and balance—as measured by the Tinetti test—showed an average improvement after the COVID-19 pandemic, functional independence, assessed using the Barthel Index, experienced a notable decline. The increased variability observed in both tests indicates a heterogeneous response to the impact of COVID-19, with some individuals showing improvement and others experiencing significant deterioration. These findings underscore the importance of implementing rehabilitation strategies tailored to individual needs in order to mitigate the functional sequelae of COVID-19 in this population.

The COVID-19 pandemic has had a profound impact on the lives of older adults, particularly in terms of physical activity and functional independence [18,19,20,21]. Reduced physical activity emerged as one of the most significant consequences of lockdowns and mobility restrictions. Recent studies have shown that the pandemic led to a marked decrease in physical activity levels among older individuals, directly correlating with increased fall risk. Physical inactivity not only affects balance and muscle strength—critical parameters assessed by the Tinetti scale [14]—but can also accelerate the loss of bone and muscle mass, thereby exacerbating fall risk and deteriorating overall health.

The observed increase in Tinetti scores post-pandemic is indeed counterintuitive and warrants careful interpretation. Possible explanations include selection bias (e.g., exclusion due to mortality of more severely impaired individuals during the pandemic), increased variability in responses reflecting differential access to rehabilitation services, or heightened attention and care provided after lockdown restrictions. Further research is needed to clarify these mechanisms.

Although BMI did not show significant influence on functional recovery in our study, it is important to recognize the limitations of BMI as an indicator of functional reserve in older populations. BMI may not adequately reflect body composition nuances, particularly in cases of sarcopenic obesity, where muscle mass reduction accompanied by fat accumulation could significantly affect functional outcomes despite normal BMI values.

For instance, a longitudinal study conducted in Italy during the pandemic found that 67% of older adults reported a decrease in daily physical activity, which correlated with lower Tinetti scores [3]. This pattern of reduced activity may have been even more pronounced in individuals with preexisting limitations or frail health conditions, who are at higher risk of falls. The lack of access to outdoor spaces, gyms, and community-based exercise programs further aggravated the challenge of maintaining an active lifestyle.

This study has several limitations that must be acknowledged. First, due to the observational and retrospective nature of the data collection, detailed information regarding acute hospital admissions or medication changes during the study period was not systematically recorded. Both factors could significantly influence functional outcomes. Additionally, the relatively long interval of approximately 3.5 years between assessments could itself contribute independently to changes in functionality and mobility, thus limiting our ability to attribute observed changes exclusively to the COVID-19 pandemic. Future studies should consider these variables explicitly to provide more robust conclusions. Another limitation of this study is the absence of systematic information regarding participation in rehabilitation programs, which may have significantly influenced functional outcomes. Additionally, detailed information on participants’ comorbidities was not available, limiting our ability to control for potential confounders. Future research should explicitly account for these variables to strengthen the validity of conclusions. An additional limitation of our study is that physical activity levels were not directly measured with specific instruments but inferred indirectly from performance on functional assessments.

Simultaneously, functional independence in activities of daily living, as measured by the Barthel Index [16], was also negatively affected. The disruption of rehabilitation and support services, along with limited social interaction, contributed to increased functional dependence [22]. During the pandemic, many older adults had to manage daily tasks without their usual support systems or community services, posing an additional challenge to maintaining independence. A study conducted in Spain reported that during lockdown periods, 45% of elderly participants experienced a significant decline in functional ability, as reflected in Barthel scores [16]. This increase in dependency not only affects quality of life but may also have long-term consequences on physical and mental health, further elevating fall risk and the likelihood of other adverse events. Moreover, heightened anxiety and social isolation—which intensified during the pandemic—may negatively impact both motivation and ability to perform basic and complex daily activities.

From a clinical standpoint, our findings emphasize the necessity for clinicians to perform periodic and comprehensive functional assessments of institutionalized elderly patients, particularly after disruptive events such as a pandemic. Clinicians should also consider integrating flexible rehabilitation programs that address both physical and psychological dimensions, even in situations of prolonged isolation or restricted access, employing both face-to-face and remote interventions to maintain or restore patient independence and overall quality of life.

Compared with pre-pandemic literature, the relationship between physical activity and fall prevention is well established. However, the pandemic created a unique context in which following these recommendations became particularly difficult, as reflected in the findings of this study. Social isolation and anxiety have been associated with increased fall risk—an association likely amplified during the pandemic. These findings emphasize the need to adapt geriatric interventions to address not only physical recovery but also the psychological and social dimensions of health in older adults.

Additionally, emerging post-pandemic evidence points to the importance of stricter pharmacological monitoring in older adults. This is due to the increased likelihood of adverse drug interactions, especially under prolonged sedentary conditions, which may significantly increase fall risk in this population [23,24].

The findings also suggest that BMI does not significantly impact functional recovery, as measured by the Tinetti and Barthel tests [25,26,27]. The absence of relevant differences between BMI groups may indicate that other variables—such as baseline clinical condition, physical activity level, or access to rehabilitation—are more critical determinants of post-COVID-19 recovery. This is consistent with prior studies that also found BMI not to be a strong predictor of functional recovery following acute illness [27].

From a clinical perspective, the results of this study suggest that post-pandemic intervention programs should aim not only to restore lost functionality but also to address new needs arising from the pandemic context. These programs should incorporate adapted physical rehabilitation interventions and reinforce community support services to regain independence. Furthermore, the limitations of this study—such as the potential impact of mental health status on outcomes—should not be overlooked. Increased levels of depression and anxiety may affect both balance and motivation to engage in daily activities, suggesting that future interventions should adopt a holistic approach, addressing both mental and physical health.

Finally, it is crucial to conduct further research evaluating the effectiveness of remote or virtual interventions aimed at maintaining or improving the physical and functional status of older adults. These modalities may prove to be more accessible and less disruptive in future health crises or similar scenarios.

## 5. Conclusions

The COVID-19 pandemic has had a diverse impact on the functionality of older adults, with a noticeable improvement in balance and gait, as measured by the Tinetti test, but a decline in functional independence, as assessed by the Barthel Index. The variability in these outcomes suggests a heterogeneous response to the pandemic, likely influenced by differences in access to and quality of rehabilitation services, as well as pre-existing health conditions.

Body Mass Index (BMI) did not appear to be a determining factor in functional recovery, indicating that other elements—such as prior physical activity levels and rehabilitation support—may play a more significant role. From a clinical perspective, these findings highlight the need for intervention programs that address not only physical recovery but also psychological and social dimensions, considering isolation and anxiety as limiting factors.

Further research is essential to explore how rehabilitation strategies can be adapted in pandemic contexts, with a focus on accessible and effective solutions to improve the functional status of older adults. These findings underscore the importance of adopting a comprehensive approach to support recovery and enhance overall well-being in this vulnerable population.

## Figures and Tables

**Table 1:** Demographic data.

	All Participants	Woman	Men	*p*-Value
	(*n* = 445)	*n* = 297	*n* = 148	
Age (years)	83.74 ± 11.42	86.07 ± 9.93	79.07 ± 12.74	<0.001 *
Height (cm)	160.40 ± 14.10	157.22 ± 11.04	167.77 ± 11.76	<0.001 *
Weight (kg)	68.31 ± 15.55	66.05 ± 14.66	72.85 ± 16.31	<0.001 *
Body mass index (kg/m^2^)	26.75 ± 6.93	26.99 ± 6.77	26.26 ± 7.23	0.27
Underweight (*n*/%)	38 (8.54%)	20 (6.73%)	18 (12.16%)	0.19
Normal (*n*/%)	158 (35.51%)	110 (37.04%)	48 (32.43%)	
Obesity (*n*/%)	119 (26.74%)	83 (27.95%)	36 (24.32%)	
Overweight (*n*/%)	130 (29.21%)	84 (28.28%)	46 (31.08%)	

* indicates statistically significant values for the defined confidence interval.

**Table 2:** Paired Sample *t*-test.

Measurement 1	Measurement 2	W	z	gl	*p*	Rank–Biserial Correlation	ES Rank–Biserial
Tinetti Pre-COVID	Tinetti Post-COVID	18,171.00	2.79		<0.005	0.21	0.07
Barthel Pre-COVID	Barthel Post-COVID	38,116.50	10.34		<0.001	0.69	0.07

Note. Wilcoxon signed-rank test.

## Data Availability

Link to datasheet… https://mailucv-my.sharepoint.com/:x:/g/personal/javier_torralba_ucv_es/ETzTXhpS8KFNlK9kjrIGBTgB-S8kGz2ND-0LWEh4bV72_A?e=zoBluH&nav=MTVfezA4MjdFNjU4LUFCOTYtQjk0RS1BMkZBLTQyRUM2RDE1MDFERn0 (accessed on 20 April 2025).
